# Isolation and propagation of a *Spiroplasma* sp. from Slovakian *Ixodes ricinus* ticks in *Ixodes* spp. cell lines

**DOI:** 10.1016/j.ttbdis.2015.05.002

**Published:** 2015-07

**Authors:** Lesley Bell-Sakyi, Ana M. Palomar, Maria Kazimirova

**Affiliations:** a The Pirbright Institute, Ash Road, Pirbright, Woking, Surrey GU24 0NF, UK; b CIBIR, C/Piqueras, 98, Logroño 26006, La Rioja, Spain; c Institute of Zoology, Slovak Academy of Sciences, 84506 Bratislava, Slovakia

**Keywords:** Spiroplasma, Tick, *Ixodes ricinus*, Tick cell line

## Abstract

*Ixodes* spp. ticks are known to occasionally harbour spiroplasmas – helical mycoplasmas in the class Mollicutes; a previous study in Slovakia reported an overall prevalence of *Spiroplasma ixodetis* of 3% in *Ixodes ricinus*. In the present study, extracts of unfed adult *I. ricinus* ticks collected from vegetation in south-western Slovakia were added to a panel of cell lines derived from *I. ricinus* and *Ixodes scapularis* embryos. The cultures were monitored by preparation and examination of Giemsa-stained cytocentrifuge smears at intervals over the subsequent 16–18 months. *Spiroplasma*-like microorganisms were detected in cultures of both tick species after 2–3 months and subcultured onto fresh, uninfected cells of the appropriate cell line up to seven times. Molecular analysis using PCR assays targeting fragments of the 16S rRNA, ITS and *rpoB* genes confirmed the identity of the microorganisms as a *Spiroplasma* sp., with between 98.9% and 99.5% similarity to *S. ixodetis*. The sequences of the spiroplasmas isolated from three different pools of ticks collected on two different occasions were identical for all three genes tested.

## Introduction

Spiroplasmas are helical mycoplasmas that infect plants and/or arthropods and may be pathogenic or commensal. Several species of ixodid ticks have been reported to harbour spiroplasmas, including *Haemaphysalis leporispalustris* (Clark, 1964; Tully et al., 1977), *Ixodes pacificus* (Tully et al., 1981, 1995), *Dermacentor marginatus* and *Ixodes ricinus* (Hornok et al., 2010). Using a PCR assay targeting the *Spiroplasma rpoB* gene, Subramanian et al. (2012) detected a *Spiroplasma* that they identified as *Spiroplasma ixodetis* in 1/52 nymphal and 1/28 adult questing *I. ricinus* ticks collected in the Podunajske Biskupice area of south-western Slovakia. Tick-borne spiroplasmas can be cultivated *in vitro* in axenic culture (Tully et al., 1977), and tick cell lines were used successfully to isolate *S. ixodetis* from *I. pacificus* ticks (Yunker et al., 1987). Henning et al. (2006) reported isolation in mammalian cells (African green monkey kidney cell line BGM) of a *Spiroplasma* sp. from a pool of *Ixodes* ticks collected from a roe deer in Germany; the *Spiroplasma* was cultured for 10 weeks and sequencing of a fragment of the 16S rRNA gene revealed that it was closely related to *S. ixodetis*.

In the present study, unfed adult *I. ricinus*, a widespread tick species that parasitises a wide range of vertebrate hosts and frequently attacks humans, were collected on two different occasions from vegetation in the campus of the Slovak Academy of Sciences (SAS) in Bratislava, Slovakia. Tick tissues were inoculated into a panel of *Ixodes* spp. tick cell lines with the aim of isolating and propagating microorganisms present in the ticks. Here we report isolation, prolonged *in vitro* cultivation and partial characterisation of a *Spiroplasma* sp. from the Slovakian ticks.

## Materials and methods

### Ticks

Unfed adult male and female *I. ricinus* ticks were collected by flagging from vegetation in the campus of the SAS, Bratislava, Slovakia, 48.17° N, 17.07°E, altitude circa 190 m above sea level, in April and June 2013. The SAS campus is a fenced area of 32 ha located on the south-western foothills of the Small Carpathians. Patches of the original oak-hornbeam forest with admixture of beech, ash, black locust, maple, limetree, elm, alder, common hazel and elder are fragmented by roads, pavements and built-up areas. Twenty-one male and 19 female ticks were collected in April 2013, and 19 male and 26 female ticks were collected in June 2013. Following microscopic examination to confirm species identity, the ticks were transferred to The Pirbright Institute where they were incubated at 15 °C, 100% relative humidity and processed within 9 days of receipt. Batches of male or female ticks were surface-sterilised by immersion in a 0.1% aqueous solution of benzalkonium chloride for 5 min and 70% ethanol for 1 min, followed by 2 rinses in sterile deionised water. The ticks were allowed to dry on sterile filter paper in a petri dish and then immersed as pools of 4–11 ticks in 1–2 ml Hanks balanced salt solution (HBSS). Using a sterile scalpel blade and watchmakers forceps, the ticks were cut into several pieces and as much of their internal organs separated from the exoskeleton as possible using the forceps and pressure from the flattened end of a glass rod. The tissue suspension was collected by pipetting, leaving as much of the exoskeleton behind as possible, and inoculated into tick cell lines as described below.

### Tick cell lines

The *I. ricinus* embryo-derived cell lines IRE/CTVM19, IRE/CTVM20 and IRE11, and the *Ixodes scapularis* embryo-derived cell lines IDE2, IDE8, ISE6 and ISE18 were grown at 28 °C or 32 °C in sealed, flat-sided culture tubes (Nunc) in 2.2 ml L-15 (Leibovitz)-based media supplemented as shown in Table 1. Cultures were inoculated with 0.2–0.3 ml of *I. ricinus* tissue suspension, incubated at the appropriate temperature for the respective cell line. Medium was changed weekly by removal and replacement of 1.5 ml medium; cultures were monitored weekly by inverted microscope for presence of contamination, and at 2–8 week intervals from day 14–22 post inoculation (p.i.) by preparation and examination of Giemsa-stained cytocentrifuge smears. When presence of putative tick-borne microorganisms was detected by microscopy, supernatant medium was passaged onto fresh cultures of the same cell line and monitoring continued as above.

### Molecular detection and identification of *Spiroplasma* species

Aliquots of 1 ml of culture suspension were processed for DNA extraction using a DNeasy Blood and Tissue Kit (Qiagen) following the manufacturer's instructions for cultured cells. Presence and initial identification of bacteria was assayed using a pan-bacterial PCR targeting a 528 bp fragment of the 16S rRNA gene (Benson et al., 2004) as described previously (Alberdi et al., 2012).

Specific PCRs targeting the 16S-23S rRNA intergenic transcribed spacer (ITS; 16S-F-MYC & 23S-R1-MYC primers; 600–1000 bp) (Volokhov et al., 2006) and a fragment of the RNA polymerase beta subunit gene (*rpoB*; RpoBF3 & RpoBR2 primers; 1443 bp) (Haselkorn et al., 2009) of *Spiroplasma* were carried out as described by the respective authors. In addition, a pan-bacterial PCR targeting a longer fragment of the *Spiroplasma* 16S rRNA gene (fD1 & rP2 primers; 1500 bp) (Weisburg et al., 1991), incorporating the sequence detected by the PCR of Benson et al. (2004), was performed on the samples that were positive in the *Spiroplasma*-specific PCRs. A negative control containing water instead of template DNA was included in all PCRs. Positive PCR products of, or close to, the expected size were purified using a High Pure PCR Product Purification kit (Roche Life Science) following the manufacturer's instructions. Amplification products were sequenced in the forward and reverse directions, and homology searches were performed in the NCBI database using the BLAST search programme (http://blast.ncbi.nlm.nih.gov/Blast.cgi). Sequences were aligned using the European Bioinformatics Institute multisequence software ClustalW2 (http://www.ebi.ac.uk/Tools/msa/clustalw2) for multiple sequence alignment. Phylogenetic analyses were conducted using *MEGA* version 6 (www.megasoftware.net). A phylogenetic tree was constructed by the neighbour-joining method. Confidence values for individual branches of the resulting tree were determined by bootstrap analysis with 1000 replicates. The evolutionary distances were computed using the maximum composite likelihood method.

## Results

Extracts of 8 pools of 4–7 male ticks and 8 pools of 4–11 female ticks were inoculated into a total of 44 cultures from one or more tick cell lines (Table 2). Of these, 37 were lost to gross bacterial and/or fungal contamination between 6 and 200 days p.i. Subculture series were initiated from surviving cultures between 69 and 116 days p.i.; between two and seven subcultures were carried out (Table 2). Between 478 and 533 days p.i., all seven surviving culture series were harvested, parent cultures (if surviving) and their subcultures were pooled, aliquots were cryopreserved in liquid nitrogen with 10% DMSO, and DNA was extracted from 1 ml of pooled culture suspension.

**Fig. 1:**
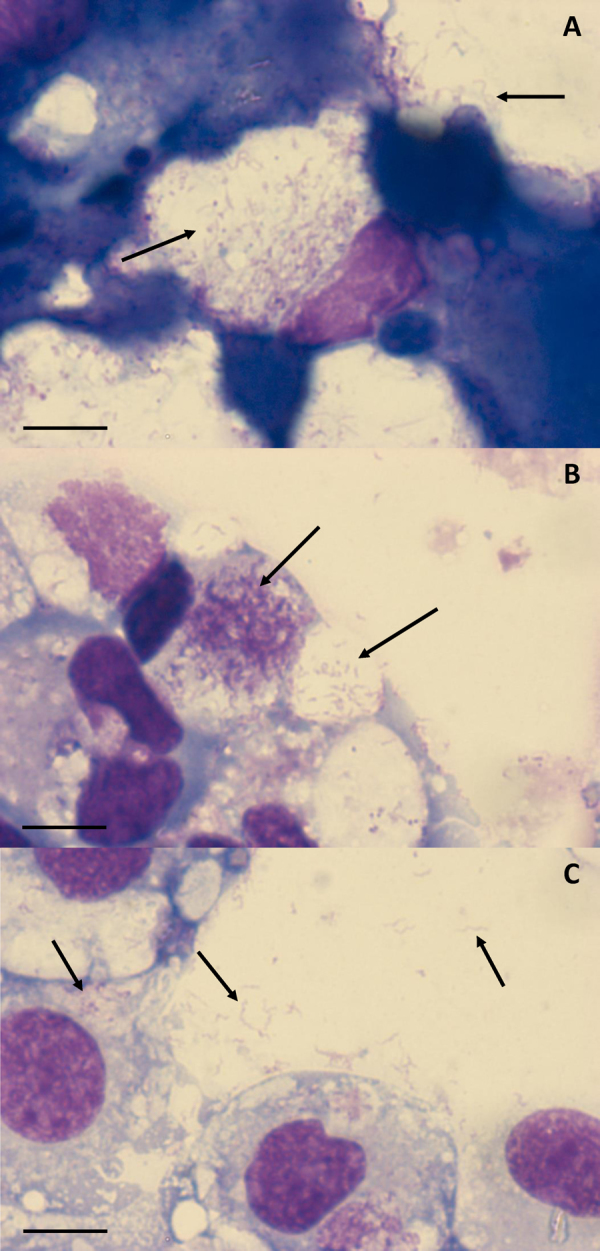
Giemsa-stained cytocentrifuge smears of *Ixodes*-derived cell lines inoculated with *Ixodes ricinus* tissues. (A) *I. ricinus* IRE/CTVM19 cells 533 days p.i.; (B) *Ixodes scapularis* IDE2 cells 527 days p.i.; (C) *I. ricinus* IRE11 cells 478 days p.i. Arrows indicate spiroplasmas. Scale bars = 10 μm.

Spiroplasmas were first identified in Giemsa-stained smears (Fig. 1) from three of the parent cultures in the cell lines IRE/CTVM19, IRE11 and IDE2 after 2–3 months *in vitro* (Table 2). Most of the spiroplasmas were located within intracellular vacuoles, forming more or less loosely-packed (Fig. 1A and B) or densely-packed (Fig. 1B) accumulations of thread-like and coccal organisms within cytoplasmic vacuoles. Occasionally free spiroplasmas with typical helical morphology were seen (Fig. 1C), possibly released from vacuoles during the cytocentrifugation process. *Spiroplasma* infection and growth was maintained through five subcultures in IRE/CTVM19 and IDE2 cells and seven subcultures in IRE11 cells. Presence of the *Spiroplasma* resulted in complete destruction of IDE2 cells, while some IRE11 cells and most IRE/CTVM19 cells survived the infection; the latter phenomenon appeared to result from development of tolerance by the whole culture, rather than the presence of a subpopulation of cells refractory to *Spiroplasma* infection within the cell lines. Spiroplasmas were not seen in any of the remaining cultures, including ISE18 cells originally inoculated with the same pooled material as the *Spiroplasma*-positive IRE/CTVM19 cells.

Molecular analysis of DNA extracted from the seven surviving uncontaminated cultures confirmed the presence of a *Spiroplasma* sp. in the three cultures that showed spiroplasmas in Giemsa-stained smears; sequencing of PCR products from all three cultures gave identical results for each target gene. No PCR products were amplified from any of the other four cultures by any of the PCR assays. Initial screening with the pan-bacterial 16S rRNA PCR of Benson et al. (2004) yielded 483 bp products that showed high levels of identity with published sequences of *Spiroplasma* spp. and uncultured bacteria obtained from a variety of arthropods (Table 3). The highest similarity (100%) was with an uncultured *Spiroplasma* sp. of the little housefly *Fannia manicata*, and highest similarity to a valid *Spiroplasma* species was 99.4% with *S. ixodetis* strain Y32 isolated from the tick *I. pacificus* (GenBank accession number NR104852). Similar results were obtained with the pan-bacterial PCR of Weisburg et al. (1991) targeting the longer 16S rRNA gene fragment, that yielded 1391 bp products which again showed high levels of identity with arthropod-derived *Spiroplasma* spp. and uncultured bacteria (Table 3), including 99.5% similarity to *S. ixodetis* strain Y32.

The sequences obtained with the *Spiroplasma*-specific PCRs targeting the ITS (815 bp) and *rpoB* (1398 bp) gene fragments showed the highest identities (98.9% and 99.2% respectively) amongst valid *Spiroplasma* species with those from *S. ixodetis* strain Y30 (GenBank accession numbers DQ004912 and DQ313833 respectively). There are no published sequences for the ITS or *rpoB* genes of most of the uncultured *Spiroplasma* spp. for which 16S rRNA sequences are available (Table 3); an exception is the *Spiroplasma* sp. detected in the planthopper *Laodelphax striatellus* for which the *rpoB* sequence (GenBank accession number AB797266) showed 99.5% similarity to the *rpoB* sequence obtained in the present study.

**Fig. 2:**
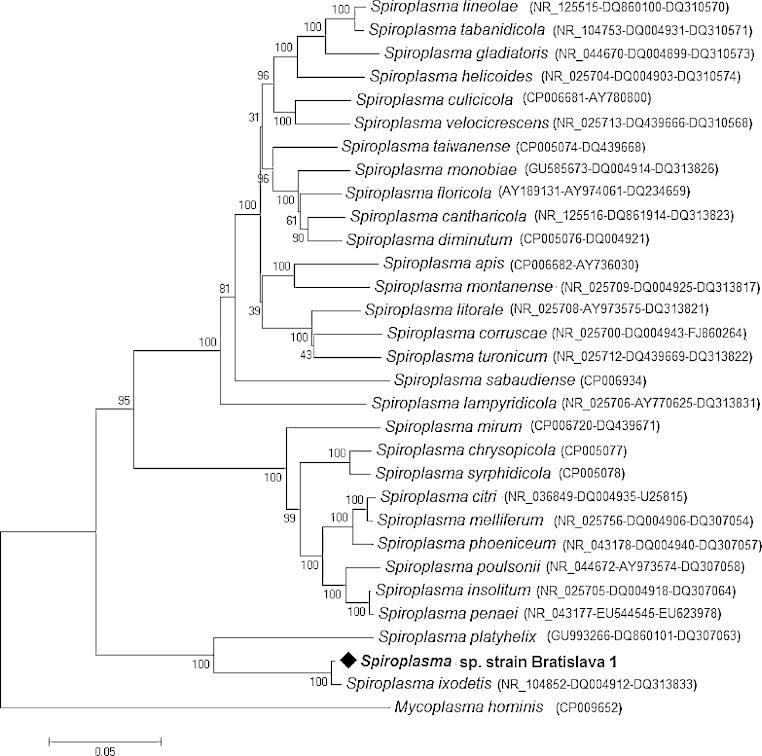
Unrooted dendrogram showing the phylogenetic position of *Spiroplasma* sp. strain Bratislava 1 (♦), isolated in the present study, among valid *Spiroplasma* species. Phylogeny is inferred from comparison of 16S rRNA (1391 bp), ITS (815 bp) and *rpoB* (1398 bp) amino-acid sequences by the neighbour-joining method (1000 replicates). *Mycoplasma hominis* is used as outgroup. GenBank accession numbers of the genes used in the comparison are shown in brackets following each *Spiroplasma* species, with multiple accession numbers separated by dashes.

The sequences obtained for the 16S rRNA (1391 bp), ITS and *rpoB* genes, from the spiroplasmas isolated in the IRE/CTVM19 cell line and designated *Spiroplasma* sp. strain Bratislava 1, were submitted to GenBank with accession numbers KP967685, KP967686 and KP967687 respectively. Table 4 shows the similarities between the partial 16S rRNA gene sequence amplified from *Spiroplasma* sp. strain Bratislava 1 in the present study and other sequences amplified from *Ixodes* spp. ticks available in GenBank. A phylogenetic tree based on the three concatenated genes (16SrRNA, ITS, *rpoB*) of *Spiroplasma* sp. strain Bratislava 1 presents the relationship between them and those of other *Spiroplasma* spp. (Fig. 2), and shows that the *Spiroplasma* sp. isolated in the present study belongs to the same clade as *S. ixodetis*.

## Discussion

Several ixodid tick genera and species have previously been reported to harbour spiroplasmas, but actual microorganisms have only been isolated from *H. leporispalustris* (*Spiroplasma mirum*, Clark, 1964), *I. pacificus* (*S. ixodetis*, Tully et al., 1977; Yunker et al., 1987) and an unspecified *Ixodes* species in Germany (*Spiroplasma* sp., Henning et al., 2006). All other reports depended on molecular detection of *Spiroplasma* DNA sequences in tick extracts, for example Taroura et al. (2005), Hornok et al. (2010), Tveten and Sjastad (2011), Subramanian et al. (2012) and Qiu et al. (2014). In the present study, a *Spiroplasma* sp. with highest similarity to *S. ixodetis* was isolated from three separate pools of unfed adult *I. ricinus* ticks; the microorganism was present in both male and female ticks, grew well in cell lines derived from both *I. ricinus* and *I. scapularis* at 28 °C or 32 °C and could be maintained *in vitro* with serial passage in tick cells for over 500 days.

As in the present study, all previously-described tick-borne spiroplasmas were isolated either from nymphal or adult ticks (Clark, 1964; Tully et al., 1977, 1995; Yunker et al., 1987) or from ticks of unspecified developmental stage but feeding on a roe deer (Henning et al., 2006). Therefore it was not possible to determine whether these spiroplasmas originated from a current or previous bloodmeal, or were transovarially-transmitted tick endosymbionts. However, spiroplasmas have also been isolated from primary cell cultures of embryonic tissues of *I. ricinus* and *Dermacentor reticulatus* ticks originating from The Netherlands (author's unpublished results), indicating that in these cases the spiroplasmas were transovarially transmitted.

Considering the ease and frequency with which spiroplasmas can be isolated from field ticks, it is surprising that there are not more records of their presence in field tick samples. The low PCR-based prevalence of 3% reported for Slovakian *I. ricinus* ticks by Subramanian et al. (2012), combined with the time taken for spiroplasmas to appear in Giemsa-stained smears in the present study, suggests that they may be present at very low densities in intact ticks. In the present study, spiroplasmas were detected in surviving cultures derived from three out of six tick pools; the positive pools comprised 4, 6 and 8 ticks each, giving a minimum prevalence in positive pools of 3/18 or 16.7% and a minimum prevalence overall of 3/31 or 9.7%. It is possible that tick cell culture isolation is a more sensitive, albeit much slower, test for *Spiroplasma* infection in ticks than molecular detection by PCR amplification. The 16S amplicon pyrosequencing approach of Qiu et al. (2014) indicated a much higher prevalence of *Spiroplasma* in Japanese *Ixodes ovatus*, but the difference between the results for this species and for *I. ricinus* could be due to different methodology, different infection rates or a combination of both.

The *Spiroplasma* sp. isolated in the present study appears to be closely related to *S. ixodetis* isolated from North American *I. pacificus* ticks (Tully et al., 1995). Similarly closely-related spiroplasmas have been detected in other tick species including European *I. ricinus* from Slovakia (Subramanian et al., 2012) and *Ixodes* sp. from Germany (Henning et al., 2006). Furthermore, *Spiroplasma* spp. closely related to *S. ixodetis* have been reported from several insect groups, including planthoppers (*L. striatellus*) (Sanada-Morimura et al., 2013), mosquitoes (*Anopheles funestus*) (Lindh et al., 2005), ladybirds (*Anisosticta novemdecimpunctata*) (Tinsley & Majerus, 2006) and mealybugs (*Antonina crawii*) (Fukatsu & Nikoh, 2000). However, those reports were based on the homology of a few gene fragments, usually a single fragment of the 16S rRNA gene. Sequence comparison of additional genes will be required to clarify the phylogenetic relationships between tick- and insect-borne spiroplasmas.

*Spiroplasma* spp. are increasingly being recognised as playing a role in human and animal disease. The first tick-derived spiroplasma to be discovered, *S. mirum*, has long been known to induce cataract formation in experimentally-infected suckling mice (Clark, 1964). More recently, a *Spiroplasma* closely related to mosquito-borne *Spiroplasma taiwanense* was found in the conjunctiva of a premature baby that acquired unilateral cataract with anterior uveitis (Lorenz et al., 2002), and *Spiroplasma turonicum* was implicated as the causal agent of a systemic human infection in Spain (Aquilino et al., 2015). Furthermore, spiroplasmas have been linked with transmissible spongiform encephalopathy in ruminants and humans (Bastian, 2014). While the aforementioned presence of spiroplasmas in tick eggs indicates that these microorganisms are natural endosymbionts of at least two European tick species, the putative role of ticks in transmission of these spiroplasmas to vertebrate hosts, and the potential infectivity and pathogenicity of tick-borne spiroplasmas other than *S. mirum* for vertebrates, require further investigation. Such studies will be greatly facilitated by the ability to isolate spiroplasmas from all developmental stages of field ticks into tick cell lines and to cultivate the spiroplasmas therein over prolonged periods and to high numbers, as demonstrated by the present study.

## Figures and Tables

**Table 1:** *Ixodes* spp. embryo-derived cell lines used in this study.

Tick species	Cell line	Incubation temperature (°C)	Culture medium	Reference
*Ixodes ricinus*	IRE/CTVM19	28	L-15^a^	Bell-Sakyi et al. (2007)
	IRE/CTVM20	28	L-15/L-15B^b^^,^^d^	Bell-Sakyi et al. (2007)
	IRE11	32	L-15B300^c^	Simser et al. (2002)

*Ixodes scapularis*	IDE2	32	L-15B300	Munderloh et al. (1994)
	IDE8	32	L-15B	Munderloh et al. (1994)
	ISE6	32	L-15B300	Kurtti et al. (1996)
	ISE18	32	L-15B300	Munderloh et al. (1994)

a L-15: L-15 (Leibovitz) medium supplemented with 10% tryptose phosphate broth (TPB), 20% foetal bovine serum (FBS), 2 mM l-glutamine (l-glut), 100 units/ml penicillin and 100 μg/ml streptomycin (pen/strep).

b L-15B: L-15B medium (Munderloh and Kurtti, 1989) supplemented with 10% TPB, 5% FBS, 0.1% bovine lipoprotein (MP Biomedicals), l-glut and pen/strep.

c L-15B300: L-15B medium diluted 3:1 with deionised water, supplemented with 10% TPB, 5% FBS, 0.1% bovine lipoprotein (MP Biomedicals), l-glut and pen/strep.

d A 1:1 mixture of the two complete media.

**Table 2:** Inoculation of tick cell lines with pooled *Ixodes ricinus* extracts. Only those pools from which at least one surviving culture was obtained are shown. The results of PCR analysis of the seven surviving cultures are shown with the day of final sampling and highest passage level (p) achieved.

Tick origin	Pool composition	Cell lines inoculated	Result
Collected 16th–17th April 2013, processed 6th May 2013	4 males	IRE/CTVM19	PCR negative, day 533, p4
	4 males	ISE6	PCR negative, day 533, p4
	5 males	IDE8	PCR negative, day 533, p2

Collected 16th–17th April 2013, processed 12th May 2013	4 males	IRE/CTVM19	*Spiroplasma* positive, day 527, p5
		ISE6	Contaminated
		ISE18	PCR negative, day 527, p2
		IDE8	Contaminated
	8 females	IRE/CTVM19	Contaminated
		IRE/CTVM20	Contaminated
		IRE11	Contaminated
		ISE6	Contaminated
		IDE2	*Spiroplasma* positive, day 527, p5
		IDE8	Contaminated

Collected 19th–20th June 2013, processed 30th June 2013	6 males	IRE/CTVM19	Contaminated
		IRE/CTVM20	Contaminated
		IRE11	*Spiroplasma* positive, day 478, p7
		ISE6	Contaminated

**Table 3:** Levels of identity between the 16SrRNA sequences obtained in the present study and published sequences from *Spiroplasma* spp. and uncultured bacteria detected in ticks and other arthropods.

Bacterium	Arthropod host	GenBank accession no.	% Identity (bp)	
			Short fragment^a^	Long fragment^b^
*Spiroplasma* sp.	*Fannia manicata*	AY569829	100 (483/483)	100 (1391/1391)
Uncultured bacterium	*Ctenocephalides felis*	EF121346	100 (483/483)	99.8 (1388/1391)
*Spiroplasma* sp.	*Anisosticta novemdecimpunctata*	AM087471	99.8 (482/483)	99.9 (1389/1391)
*Spiroplasma* sp.	*Anopheles funestus*	AY837731	99.8 (482/483)	99.8 (1388/1391)
*Spiroplasma* sp.	*A. funestus*	AY837732	99.8 (482/483)	99.7 (1387/1391)
Uncultured bacterium	*Meimuna mongolica*	KC424774	99.8 (482/483)	99.6 (1386/1391)
Uncultured bacterium	*M. mongolica*	KC424775	99.8 (482/483)	99.6 (1385/1391)
*Spiroplasma* sp.	*Antonina crawii*	AB030022	99.6 (481/483)	99.8 (1388/1391)
*Spiroplasma* sp.	*Harmonia axyridis*	AJ132412	99.6 (481/483)	99.6 (1385/1391)
*Spiroplasma ixodetis*	*Ixodes pacificus*	NR_104852	99.4 (481/484)	99.5 (1386/1393)
*Spiroplasma* sp.	*Acyrthosiphon pisum*	AB048263	99.4 (480/483)	99.7 (1387/1391)
*Spiroplasma* sp.	*Laodelphax striatellus*	AB553862	99.4 (480/483)	99.6 (1383/1389)
*Spiroplasma* sp.	*Agathemera claraziana*	JF266577	99.4 (480/483)	99.6 (1386/1391)

a 483 bp sequence obtained using the PCR of Benson et al. (2004).

b 1391 bp sequence obtained using the PCR of Weisburg et al. (1991).

**Table 4:** Similarity of the partial 16S rRNA gene sequence of *Spiroplasma* sp. strain Bratislava 1 isolated from *Ixodes ricinus* ticks in the present study with *Spiroplasma* spp. sequences amplified from *Ixodes* spp. ticks and deposited in GenBank.

*Spiroplasma* (reference)	Tick species	GenBank accession no.	Length (bp)	No.	1	2	3	4	5	6
*Spiroplasma* sp. Bratislava 1 (this study)	*I. ricinus*	KP967685	1391	1	100					
*S. ixodetis* (ATCC:33835) (Tully et al., 1995)	*I. pacificus*	NR_104852	1451	2	99.5	100				
*Spiroplasma* sp. (Henning et al., 2006)	*Ixodes* sp.	DQ012506	219	3	98.6	98.2	100			
*Spiroplasma* sp. (Hornok et al., 2010)	*I. ricinus*	EU170606 EU170609 EU1706014 EU1706015	195	4	98.5	97.5	97.7	100		
*Spiroplasma* sp. (Hornok et al., 2010)	*I. ricinus*	EU170607 EU170608	195	5	96.4	95.4	96.6	98.0	100	
*Spiroplasma* sp. (Halos et al., unpublished, 2005)	*I. ricinus*	DQ065809	171	6	76.8	81.2	88.4	93.2	91.4	100
